# An 11-year longitudinal analysis of refracture rates and public hospital service utilisation in Australia’s most populous state

**DOI:** 10.1007/s11657-022-01105-w

**Published:** 2022-05-06

**Authors:** Jennifer Williamson, Zoe Michaleff, Francisco Schneuer, Peter Wong, Christopher Needs, Julia Thompson, Liz Hay

**Affiliations:** 1 Economics and Analysis Unit, Strategic Reform and Planning Branch, NSW Ministry of Health, St Leonards, New South Wales, Australia; 2 Institute for Evidence Based Healthcare, Faculty of Health Science and Medicine, Bond University, Robina, QLD Australia; 3 Institute for Musculoskeletal Health, School of Public Health, The University of Sydney and Sydney Local Health District, Camperdown, New South Wales Australia; 4 The Children’s Hospital at Westmead Clinical School, Faculty of Medicine and Health, The University of Sydney, Westmead, NSW Australia; 5 Department of Rheumatology, Westmead Hospital, Westmead, NSW Australia; 6 Westmead Clinical School, Faculty of Medicine, The University of Sydney, Westmead, NSW Australia; 7 Rural Medical School, Faculty of Medicine, University of New South Wales, Kensington, NSW Australia; 8 Healthy Bones Australia, Sydney, NSW Australia; 9 Department of Rheumatology, Royal Prince Alfred Hospital, Camperdown, NSW Australia; 10 Agency for Clinical Innovation Musculoskeletal Network, St Leonards, New South Wales, Australia

**Keywords:** Osteoporosis, Minimal trauma fracture, Service costs, Service utilisation, Fracture prevention

## Abstract

**Summary:**

This detailed 11-year longitudinal analysis calculated the public health cost of managing refractures in people aged ≥ 50 years in Australia’s most populous state. It provides current and projected statewide health system costs associated with managing osteoporosis and provides a foundation to evaluate a novel statewide model of fracture prevention.

**Purpose:**

The purpose of this longitudinal analysis was to calculate current and projected refracture rates and associated public hospital utilisation and costs in New South Wales (NSW), Australia. These results will be used to inform scaled implementation and evaluation of a statewide Osteoporotic Refracture Prevention (ORP) model of care.

**Methods:**

Linked administrative data (inpatient admissions, outpatient attendances, Emergency Department presentations, deaths, cost) were used to calculate annual refracture rates and refracture-related service utilisation between 2007 and 2018 and healthcare costs between 2008 and 2019. Projections for the next decade were made using ‘business-as-usual’ modelling.

**Results:**

Between 2007 and 2018, 388,743 people aged ≥ 50 years experienced an index fracture and 81,601 had a refracture. Refracture was more common in older people (rising from a cumulative refracture rate at 5 years of 14% in those aged 50–64 years, to 44% in those aged > 90 years), women with a major index fracture (5-year cumulative refracture rate of 26% in females, compared to 19% for males) or minimal trauma index fracture and those with an osteoporosis diagnosis (5-year cumulative refracture rate of 36% and 22%, respectively in those with and without an osteoporosis diagnosis). Refractures increased from 8774 in 2008 to 14,323 in 2018. The annual cost of refracture to NSW Health increased from AU$130 million in 2009 to AU$194 million in 2019. It is projected that, over the next decade, if nothing changes, 292,537 refracture-related hospital admissions and Emergency Department presentations and 570,000 outpatient attendances will occur, at an estimated total cost to NSW Health of AU$2.4 billion.

**Conclusion:**

This analysis provides a detailed picture of refractures and associated projected service utilisation and costs over the next decade in Australia’s most populous state. Understanding the burden of refracture provides a foundation for evaluation of a novel statewide ORP model of care to prevent refractures in people aged ≥ 50 years.

**Supplementary Information:**

The online version contains supplementary material available at 10.1007/s11657-022-01105-w.

## Introduction

Osteoporosis is a chronic disease characterised by reduced bone density and strength, which predisposes people to minimal trauma fractures (MTFs; also known as osteoporotic or fragility fractures). While osteoporotic fracture site varies with age, the distal forearm, humerus, hip and spine are the commonest MTF sites. Such fractures result in significant pain, reduced mobility, loss of function and reduced quality of life. MTFs predict future fracture [1, 2] and are associated with increased mortality [3]. In the Dubbo Osteoporosis Epidemiology Study, 51% of men and 39% of women died within 5 years of a fracture [4] and up to one in five people with a hip fracture died within 12 months [3].

Osteoporotic fractures are a major and growing public health problem worldwide due to the ageing population and associated morbidities that increase fracture risk [5–7]. In Australia, MTFs affect one in four men and two in five women aged 50 years and older [8]. Despite the widespread availability of effective anti-resorptive medications [9, 10] that lower fracture risk and mortality [11], osteoporosis remains undertreated, despite MTFs identifying those at risk [12–14].

Healthcare in Australia is delivered by a mix of public, private and non-government service providers, with public healthcare underpinned by Medicare, Australia’s universal health insurance scheme [15]. In New South Wales (NSW), Australia’s most populous state, the public health system comprises 228 public hospitals and facilities across 17 NSW Health Local Health Districts and Specialty Networks. NSW Health employs over 100,000 staff, providing health services to a growing population of 8.1 million people across a diverse geography of over 800,000 km^2^ [16].

In NSW, the health system burden and economic cost of fracture management are significant and growing as the population ages. Fractures and refractures are seen in multiple public and private healthcare settings. Osteoporosis Australia^1^ estimated that, in 2017, the total cost of low bone density in residents of NSW and the neighbouring Australian Capital Territory older than 50 years was AU$1.1 billion, of which AU$740 million (67%) related to fracture treatment [17]. This probably underestimates the true picture due to incomplete patient follow-up and analyses restricted to re-admissions to the same hospital [18].

In 2010, NSW Health identified an urgent need for a novel, comprehensive, evidence-based approach to refracture prevention. A statewide model of care for Osteoporotic Refracture Prevention (ORP) was developed to identify and assess people at increased risk of refracture to optimise bone health. The aim was to reduce refractures by patient identification and increase patient access to bone health investigations, care coordination and treatment. This was done within the framework of four domains of value-based healthcare (health outcomes that matter to patients, the experiences of receiving care, the experiences of providing care, effectiveness and efficiency of care) [19]. The ORP model of care was piloted in 2011 and, in 2017/2018, was implemented at every Local Health District in NSW.

A critical foundation for evaluating the ORP model of care is an accurate understanding of refracture rates and associated health service utilisation in NSW. In 2019, we undertook a statewide longitudinal analysis with the primary objective of accurately identifying the refracture rate in people aged 50 years and older treated for an index (first) fracture in NSW. Secondary objectives were to estimate refracture rates over time and stratified by patient and index fracture characteristics (e.g. age, sex, osteoporosis diagnosis, public/private hospital, major/minor fracture and minimal/major trauma), and to determine current and projected public hospital service utilisation. While the ORP focuses on MTFs, statewide planning requires consideration of all refractures and associated costs.

## Methods

### Data sources

This analysis used linked administrative data on inpatient admissions, outpatient attendances, Emergency Department (ED) presentations, deaths and cost prepared by the Centre for Health Record Linkage^2^ (CHeReL) [20].

### Study population and definitions

The study population was NSW residents aged ≥ 50 years with a fracture diagnosis recorded: (a) on admission to any NSW public or private hospital or (b) on discharge without an admission record by a NSW hospital ED between 2007/2008 and 2017/2018 (financial years).^3^ Data were only available for outpatient services provided during 2015/2016 to 2017/2018 (financial years).^4^ Due to lack of available costing data during the 2007/2008 financial year, the study period for healthcare costs related to refracture was 2008/2009 to 2018/2019 (financial years).^5^

Fractures were defined by International Classification of Diseases-10-Australian Modification (ICD 10-AM) codes for fracture (including those with a principal or additional diagnosis of osteoporosis) and Systematized Nomenclature of Medicine – Clinical Terms (SNOMED-CT) codes. The analysis differentiated major and minor fractures as well as fractures arising from minimal or major trauma. See Supplementary Data for data sources.

### Index fractures

The number of index fractures was calculated by summing the total number of NSW public and private hospital admissions (acute and non-acute) and ED presentations with a recorded fracture diagnosis in people aged ≥ 50 years. The rate of index fracture was calculated using the number of NSW residents aged ≥ 50 years at corresponding timepoints. Sociodemographic and health characteristics described for people with an index fracture included age, sex, public/private hospital, Local Health District, type of fracture, osteoporosis diagnosis, dementia diagnosis and Charlson Comorbidity Index [21].

### Refractures

Refracture rates and median time to refracture (interquartile range) were calculated for people with a recorded index fracture diagnosis using hospital admission and ED presentation data. Cumulative refracture rates were calculated using Kaplan–Meier methods accounting for follow-up time and deaths and adjusted for age, sex and fracture type. Cumulative refracture rates were also stratified by variables obtained at the time of the index fracture; these were age, sex, osteoporosis diagnosis, public/private hospital, major/minor fracture and minimal/major trauma. Trends in 1- to 5-year cumulative refracture rates from year of index fracture were presented descriptively.

### Health service utilisation

Annual health service utilisation associated with refracture was calculated using the total number of public hospital admissions, ED presentations and outpatient service events. Any admission or ED presentation with a fracture diagnosis occurring within 28 days of the index fracture or refracture was considered part of the same episode of care. Any outpatient service utilisation event within 6 months of refracture was considered related to the same refracture.

### Health service costs

Annual health service costs associated with refracture used assigned National Weighted Activity Unit (NWAU) for each episode of care multiplied by the NSW State Price [22]. In this study, costs have been reported from a NSW Health perspective, e.g. costs associated with inpatient medications, pathology, imaging and Emergency admissions. Outpatient costs were not included; as in Australia, these are covered by various Federal Government funding schemes; for example, outpatient medication costs are covered by the Pharmaceutical Benefits Scheme, and outpatient imaging costs and general practitioner visits are covered by the Medicare Benefits Schedule.

### Projections

NSW Health Statistics population estimates were used to calculate 10-year projections for index fracture rates and healthcare service utilisation over the period 2019/2020 to 2028/2029 (financial years).^6^ Annual and overall projections for refracture numbers and associated health service utilisation were estimated using probabilistic health forecasting methods. Healthcare cost to NSW Health was estimated by applying the 2018–2019 NSW State Price and average NWAU to projected fracture numbers.

## Results

### Index fractures

**Fig. 1:**
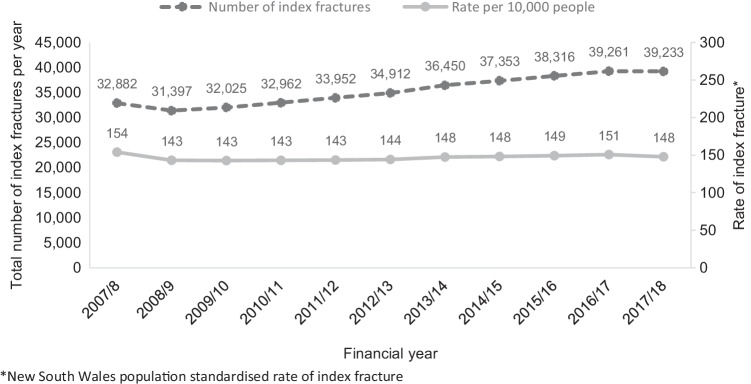
Total annual number and rate of index fractures in people aged 50 years and older residing in New South Wales over the study period 2007/2008 to 2017/2018. *New South Wales population standardised rate of index fracture

Between July 1, 2008, and June 30, 2018, there were 388,743 index fractures in people aged ≥ 50 years with an annual population-standardised rate of 143–154 per 10,000 people (Fig. 1) in NSW. Of the people with an index fracture, 33% resided in a rural Local Health District. Most index fractures (94%) were managed in NSW public hospitals and typically involved the ED, as part of an acute admission (52%) or with treatment in the ED only (41%). Only 6% of index fractures were managed in private hospitals.

Most index fractures followed minimal trauma (74%). The most common anatomic sites were the forearm (24%), lower leg/foot (18%) and hip (14%). Index fractures were categorised as a major fracture in 44% of separations (Table 1).

**Table 1:** Types of index fractures and patient characteristics for separations due to index fracture in New South Wales in people ≥ 50 years 2007/2008 to 2017/2018 (financial years)

Characteristic	Category	No. of separations (%)
Sex	Male	145,736 (38)
	Female	243,001 (63)
Type of fracture	Minor fracture	216,992 (56)
	Major fracture	171,751 (44)
Trauma	Minimal trauma	287,356 (74)
	Major trauma	101,387 (26)
Pre-existing osteoporosis diagnosis	No	344,631 (89)
	Yes	44,112 (11)
Dementia diagnosis	No	360,404 (93)
	Yes	28,339 (7)
Charlson Comorbidity Index	No comorbidities (0)	196,806 (78)
	1	30,736 (12)
	2 +	26,485 (10)

*Definitions*: minor fracture: all fractures not meeting the definition of a major fracture, based on ICD-10-AM, ICD-9 or SnomedCT codes; major fracture: single, or multiple fractures of the spine, hip, pelvis, leg and shoulder regions, based on ICD-10-AM, ICD-9 or SnomedCT codes; minimal trauma: fractures resulting from an event that would not be expected to fracture a healthy bone, based on ICD-10-AM trauma cause codes or EDDC 4 (non-urgent) and 5 (semi-urgent); major trauma: based on ICD-10-AM trauma cause codes or EDDC categories 1–3

*Abbreviations*: *ICD-10-AM*, International Classification of Diseases-10-Australian Modification; *SnomedCT*, Systematized Nomenclature of Medicine – Clinical Terms; *EDDC*, Emergency Department Data Collection

Index fractures were more common in women (63%) and over one-third (37%) were in people aged 50–64 years. A small proportion of people with an index fracture had a recorded diagnosis of osteoporosis (11%) or dementia (7%) (Table 1). Most hip fractures (97%) were treated in the admitted setting.

### Refractures

**Fig. 2:**
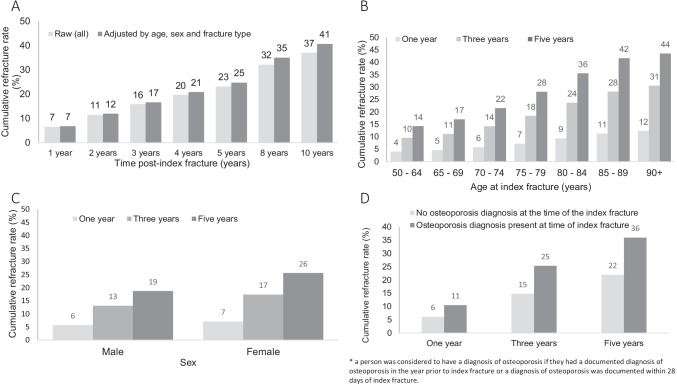
Effects of age, sex and osteoporosis diagnosis on cumulative refracture rates. Panel **A** shows adjusted and unadjusted cumulative refracture rate over time for entire cohort; panel **B** shows 1-, 3- and 5-year cumulative refracture rate stratified by age; panel **C** shows 1-, 3- and 5-year cumulative refracture rate stratified by gender; panel **D** shows 1-, 3- and 5-year cumulative refracture rate stratified by diagnosis of osteoporosis

Cumulative refracture rates for the 388,743 people with an index fracture between 2007/2008 and 2017/2018 are shown in Fig. 2A. The unadjusted refracture rate was 7% in year 1, rising to 37% at 10 years post-index fracture. When adjusted for age, sex and fracture type, refracture rates were 7% in year 1, rising to 41% at year 10 (Fig. 2A). Most refractures (91%) were managed in NSW public hospitals. The cumulative refracture rate was higher for older people, women and those with documented osteoporosis at the time of index fracture (Fig. 2B–D). Refracture rates were also higher in those with a major index fracture compared to those with a minor index fracture (5-year cumulative refracture rate 30% versus 19%), and those with a minimal trauma index fracture compared to those with a major trauma fracture (5-year cumulative refracture rate 25% versus 18%).

**Fig. 3:**
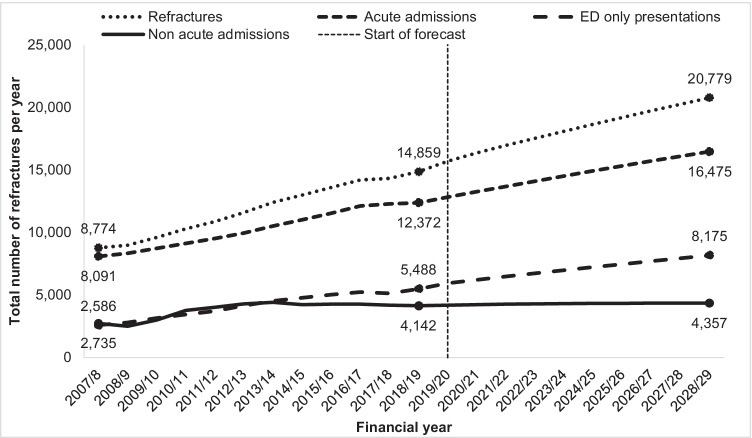
Impact of refractures on past and projected health service utilisation

The annual number of refractures increased by 63% from 8774 in 2007/2008 to 14,323 in 2017/2018 (Fig. 3). Of those who returned to hospital with a refracture during the study period, 60,831 (75%) had one refracture, 14,763 (18%) had two refractures and 6007 (7%) had three or more refractures. The median time between index fracture and refracture was 2.1 years (interquartile range 0.8–4.1 years).

### Health service utilisation

Between 2007/2008 and 2017/2018, refractures resulted in a total of 194,582 acute and non-acute admissions and ED presentations to public hospitals in NSW, equivalent to 1.8 million bed days. Over half, the health service utilisation associated with refracture was attributable to acute admissions (Fig. 3).

Between 2015/2016 and 2017/2018 — the timeframe over which outpatient data were available — a total of 108,451 refracture-related outpatient service events were delivered. Outpatient services most frequently associated with refracture were Aged Care, and Fracture and Rehabilitation Clinics (36%, 27% and 22% of 166,878 outpatient service events in 2017/2018, respectively). Only a small proportion of outpatient service events (< 4%) were for osteoporosis management or fall prevention.

### Health service costs

**Fig. 4:**
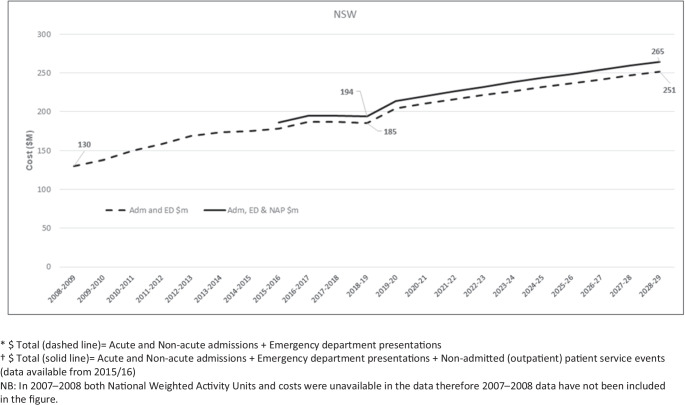
Past and projected impact of refracture on costs for New South Wales public health services. *$ Total (dashed line) = Acute and Non-acute admissions + Emergency Department presentations. †$ Total (solid line) = Acute and Non-acute admissions + Emergency Department presentations + Non-admitted (outpatient) patient service events (data available from 2015/16). NB: In 2007–2008, both National Weighted Activity Units and costs were unavailable in the data; therefore, 2007–2008 data have not been included in the figure

Using 2018/2019 constant prices, the total annual cost of refractures to the NSW public health system increased from AU$130 million in 2008/2009 to AU$194 million in 2018/2019 (including costs for outpatient services for the 2015/2016 to 2018/2019 period). The total cost of refractures over the 2008/2009 to 2018/2019 analysis period was AU$1.7 billion (Fig. 4).

### Projections

It is estimated that continuing ‘business-as-usual’ in NSW over the next decade (2019/2020 to 2028/2029) would see the following: a 40% increase in refractures (equivalent to approximately 600 more refractures per year) (Fig. 3); a total of 292,537 refracture-related acute and non-acute admissions and ED presentations to NSW public hospitals; and a total of 570,000 refracture-related public outpatient service events.

This would result in a projected 3% annual increase in total cost of refractures to the NSW public health system (approximately AU$7.1 million each year), reaching a 10-year total cost of AU$2.4 billion by 2028/2029 (Fig. 4).

## Discussion

Reducing osteoporotic fractures is a major public health priority in NSW. Inability to effectively estimate the burden of disease has been a barrier to systematic and scaled implementation of new models of care. This is the first comprehensive statewide longitudinal analysis linking multiple large population-based datasets to directly quantify the current and future burden of refracture for the public health system in Australia. While the annual population-standardised index fracture rate was stable over the study period, the absolute number of refractures and adjusted cumulative refracture rates increased. As expected, cumulative refracture rates were higher for older people, women and those with diagnosed osteoporosis at time of index fracture. Despite the opportunity for fracture prevention measures following index fracture, 75% of people had one refracture, 18% had two refractures and 7% had three or more refractures over the study period.

Without additional intervention and further scaling of the NSW Health ORP programme, a 40% increase in refractures will occur over the next decade, equivalent to approximately 600 more refractures each year. This is projected to equate to an annual increase in total cost of refractures to the NSW public health system of 3% over the next decade (about AU$7.1 million/year): a 10-year cumulative cost of AU$2.4 billion, representing around 1% of the likely total state health budget.

The Dubbo Osteoporosis Epidemiology Study, one of the longest running prospective worldwide cohorts assessing bone health, has provided many valuable insights into fracture epidemiology [3, 23, 24]. However, findings are from one cohort in a rural city. This comprehensive state-wide linkage analysis of multiple large population-based datasets has allowed analysis of Australia’s most populous state. Consistent with Dubbo Osteoporosis Epidemiology Study [24], refracture rates increased with age. However, whereas absolute refracture risk was similar for men and women in Dubbo Osteoporosis Epidemiology Study, the current study demonstrated higher cumulative refracture rates in women (Fig. 2C). The reason for this is unclear.

A MTF is a sentinel event that portents future fractures and should prompt bone health assessment and implementation of secondary preventive measures, including bone protective therapy and fall prevention strategies [25]. However, multiple studies in primary care and hospital settings show this is often not undertaken [12, 13]. Data from the Australian and New Zealand Hip Fracture Registry showed that, as recently as 2018, only 18% of those with a hip fracture were receiving bone-protective medication on discharge from Australian hospitals [26]. Consistent with these data, only 4% of the 108,451 refracture-related outpatient service events between 2015/2016 and 2017/2018 utilised outpatient services for osteoporosis management or fall prevention.

Failure to manage refractures in a systematic way will result in adverse patient experience and outcomes and place significant resource burden on the acute care system. NSW Health is currently implementing a statewide evidence-based Osteoporotic Refracture Prevention (ORP) model of care, as part of its commitment to value-based healthcare. This involves a fracture liaison service (FLS) with case finding, bone health management and follow-up coordinated by a fracture liaison coordinator (FLC), usually in collaboration with a local medical ‘champion’ [6]. This approach has been shown internationally and locally to lower refracture rates [26, 27], economic burden [28] and mortality [29].

It is too early to determine the statewide effectiveness of the ORP model of care in reducing refractures and attendant economic costs. However, a NSW Health formative evaluation of multiple early adopters of an early model of care prior to statewide implementation of ORP services found a positive impact on refracture rates [27]. Furthermore, a detailed study at a single hospital in NSW found refracture rates at 24 months post-index fracture were lower in those who attended a FLS compared with those who did not (5.1% of 214 FLS attendees vs 16.4% of 220 non-attendees; *p* < 0.001) [28]. The same group showed a 30% reduction in refracture and a 40% reduction in major refracture at a FLS hospital compared with a similar non-FLS hospital over a 3-year period [29, 30]. A recent Swedish retrospective cohort study using electronic health records from 2012 to 2017 also found risk of refracture was 18% lower in hospitals with an FLS compared to those without one and showed an 18% reduction in risk of refracture following implementation of the FLS compared with the control period [31, 32].

Defining the background rate of fracture and refracture is vital to inform and evaluate the ORP model of care in NSW — the aim of this study. Assuming implementation of the ORP model of care in NSW results in a realistic 10% reduction in refractures compared with ‘business as usual’; over the 10 years to 2028–2029, the cost avoided in NSW is estimated to reach AU$240 million. The results of our study will provide baseline parameters from which to assess whether a pragmatic 10% reduction in refractures has occurred with statewide implementation of ORP services. Should a magnitude of this reduction not occur, then other interventions will need to be trialled, for example implementation of ORP services in primary care or a public health education/advertising campaign using traditional and social media.

The current analysis exemplifies the power of data linkage. However, limitations of administrative datasets need consideration. ‘Survivor bias’ may underestimate true refracture rates and interhospital variation in quality and completeness of data coding can affect data integrity. Admission diagnosis codes do not accurately identify MTFs or differentiate between existing fractures and refractures. There is an implicit understanding that this study will underestimate the prevalence of vertebral frailty fractures due to their often-asymptomatic nature. However, work is underway in NSW to allow more targeted identification of MTFs with a statewide electronic medical record solution, including fracture diagnosis codes to allow more complete data searches. The initiation of bone protective therapy following fracture was also not assessed as this was beyond the remit of this study.

Other limitations of the current study were that outpatient data were only available for the period 2015/2016 to 2017/2018. Furthermore, outpatient service access varies across the state, and outpatient service episodes due to fall prevention or osteoporosis care may have been attributed to aged care. The analysis only included those treated in NSW hospitals; people who moved interstate, received treatment in other states or who were treated exclusively in primary care were not included. Effect of fracture on quality of life, indirect costs, productivity losses, outpatient medication costs and medication adherence were also not studied.

The analysis included refractures up to 10 years following index fracture. Refracture estimates may change with longer follow-up. There was also a 1-year difference in timeframes for activity data (2007/2008 to 2017/2018) and health service costs (2008/2018) due to incomplete costing data for the first year of activity analysis. The probable impact of these limitations is that our findings are a conservative estimate of the refracture burden on the NSW public health system.

Despite the above limitations, this study provides the first comprehensive statewide assessment of current and predicted rate of refracture and associated service utilisation in Australia’s most populous state. It provides a critical foundation from which to evaluate the effectiveness of statewide implementation of the ORP model of care — the centrepiece of the NSW Health response to this escalating public health issue. Implementation of the ORP model of care has the potential to decrease the rate of growth demand for acute care refracture treatment and prevent future refractures. Information from this longitudinal analysis will be used to shape the model of care, maintain alignment of the ORP with the principles of value-based healthcare and evaluate the outcomes and effectiveness of the ORP in NSW, including its economic analysis. Lessons learnt from this study and from future planned work evaluating statewide implementation of an ORP model of care will be of major relevance for other countries which seek to address this major public health issue.

## Supplementary Information

Below is the link to the electronic supplementary material.

Supplementary file1 (DOCX 37 KB)

## Footnotes

1 Osteoporosis Australia (now called Healthy Bones Australia) is a national non-for-profit consumer organisation focusing on fracture prevention and improving bone health across Australia.

2 The CHeReL is managed by the NSW Ministry of Health. It helps researchers, planners and policy makers access linked health data about people in the NSW and ACT using a secure data linkage system, one of the largest of its kind in Australia. See https://www.cherel.org.au/about-us

3 Financial years defined as July 1, 2007 to June 30, 2018.

4 Financial years defined as July 1, 2015 to June 30, 2018.

5 Financial years defined as July 1, 2008 to June 30, 2019.

6 Financial years defined as July 1, 2019 to June 30, 2028.
