# Supplementary material for: An 11-year longitudinal analysis of refracture rates and public hospital service utilisation in Australia’s most populous state
Source: Arch Osteoporos. 2022 May 6;17(1):76. doi: 10.1007/s11657-022-01105-w (PMC9076713; doi:10.1007/s11657-022-01105-w)
Supplement: Supplementary file 1 — Supplementary file1 (DOCX 37 KB) [file 11657_2022_1105_MOESM1_ESM.docx]

**An 11-year longitudinal analysis of refracture rates and public hospital service utilisation in Australia’s most populous state**

**SUPPLEMENTARY DATA: Data sources and definitions**

| **Data source** | | **Date** |
| --- | --- | --- |
| NSW Admitted Patient Data Collection (APDC), census of public and private hospital separations | | 1 July 2007 to 30 June 2018 |
| NSW Emergency department Data Department Data Collection (EDDC) | | 1 July 2007 to 30 June 2018 |
| NSW Hospital Performance Dataset (HOPED) | | 1 July 2007 to 30 June 2018 |
| NSW Non-admitted Patient Data Collection (e.g. relevant Leading Better Value Care clinics including fracture clinic, rehabilitation (sub-acute and non-acute patient) data) | | 1 July 2015 to 30 June 2018 |
| NSW Registry of Births, Deaths and Marriages | | 1 July 2007 to 30 June 2018 |
| Register of Outcomes, Value and Experiences (ROVE) | | 1 July 2007 to 30 June 2018 |
| Activity Based Funding – national weighted activity units (NWAU) across all care settings | | 1 July 2008 to 30 June 2019 |
| **Definitions** | | |
| Major fractures | Single, or multiple fractures of the spine, hip, pelvis, leg and shoulder regions | |
| Minor fractures | All fractures not meeting the definition of a major fracture | |
| Minimal trauma fractures | Fractures resulting from an event that would not be expected to fracture a healthy bone; based on ICD-10-AM trauma cause codes or EDDC 4 (non-urgent) and 5 (semi-urgent) | |
| Fractures resulting from major trauma | Based on ICD-10-AM trauma cause codes or EDDC categories 1–3 | |
